# Distribution of Rotavirus Genotypes Circulating in Ahvaz, Iran in 2016

**DOI:** 10.22034/ibj.22.2.107

**Published:** 2018-03

**Authors:** Azarakhsh Azaran, Manoochehr Makvandi, Ali Teimoori, Saeedeh Ebrahimi, Farzad Heydari, Roya Nikfar

**Affiliations:** 1 Infectious and Tropical Diseases Research Center, Health Research Institute, Ahvaz Jundishapur University of Medical Sciences, Ahvaz, Iran; 2 Virology Department, Ahvaz Jundishapur University of Medical Sciences, Ahvaz, Iran; 3 Cukurova University, Medicine Faculty, Medical Microbiology Department, Adana, Turkey; 4 Department of Infectious Diseases, Aboozar Children’s Hospital, Ahvaz Jundishapur University of Medical Sciences, Ahvaz, Iran

**Keywords:** Rotavirus, Genotype, Emergence, Iran

## Abstract

**Background:**

Group A rotavirus (RVA) mainly causes acute gastroenteritis, exclusively in young children in developing countries. The prevalence and determination of the molecular epidemiology of rotavirus genotypes will determine the dominant rotavirus genotypes in the region and will provide a strategy for the development of appropriate vaccines.

**Methods:**

A total of 100 fecal samples were collected from children below five years with acute gastroenteritis who referred to Aboozar Children’s Hospital of Ahvaz city during October 2015 to March 2016. All samples were screened by latex agglutination for the presence of rotavirus antigen. Rotavirus-positive samples were further analyzed by the semi-multiplex RT-PCR, and the sequencing was performed for G/P genotyping.

**Results:**

Findings showed that 32% of the specimens were RVA-positive. Among the 32 *VP7* genotyped strains, the predominant G genotype was G9 (37.5%), followed by G2 (21.9%), G1 (12.5%), G12 (9.4%), G4 (9.4%), G2G9 (6.3%), and G3 (3.1%). Among the 31 VP4 genotyped strains, P[8] genotype was the dominant (62.5%), followed by P[4] (31.3%) and P[4] P[8] (3.1%). The genotypes for G and P were identified for 31 rotaviruses (96.87%), but only one strain, G9, remained non-typeable for the P genotype. The most prevalent G/P combination was G9P[8] (28.5%), followed by G2P[4] (18.8%), G1P[8] (9.4%), G12P[8] (9.4%), G4P[8] (9.4%), G2G9P[4] (6.3%), G9P[4] P[8] (3.1%), G3P[8] (3.1%), G9P[4] (3.1%), G2P[8] (3.1%), and G9P[non typeable] (3.1%).

**Conclusion:**

A novel rotavirus strain, G12, was detected, for the first time, in patients from the southwest of Iran. Comprehensive investigations are required to evaluate the emergence of this strain.

## INTRODUCTION

Pediatric diarrhea is frequently lethal since this illness causes severe dehydration[1]. There are multiple causes of the disease, including bacterial, parasitic and viral infections[2-4]. Viruses, specifically of the rotavirus group A, are the predominant factors of viral gastroenteritis in children aged <5 years worldwide[5]. Rotaviruses have high frequency rates of morbidity and mortality in developed and developing countries, respectively[6]. These viruses are transmitted via the fecal-oral route, which can happen directly from person to person and through contaminated drinking water[7]. It has been estimated that about 125 million cases of diarrhea and more than 453,000 deaths occur annually in the world due to gastroenteritis caused by rotaviruses[8-10].

Rotaviruses are non-enveloped, double-stranded RNA viruses belonging to the Reoviridae family. The genome comprises 11 segments that encode six structural proteins (*VP1-VP4*, *VP6*, and *VP7*) and six non-structural proteins (*NSP1-NSP5*/*NSP6*). The serologic cross-reactivity of the middle layer protein, *VP6*, has firmly identified seven serogroups (A-G); however, based on *VP6* genetic diversity, there are likely at least eight serogroups (A-H)[11-13]. The majority of human rotavirus infections belong to group A, though some strains of rotaviruses in groups B and C can also cause diarrhea in humans.

Rotaviruses are classified based on their serological characteristics or the genetic diversity of two outer capsid proteins, *VP7* (glycosylated, G-type) and *VP4* (protease sensitive, P-type)[14], since these protein targets for antibodies are important for broadening vaccine[15]. So far, 27 different G- and 37 different P-genotypes have been identified, and approximately, 73 G/P genotypes of group A rotavirus have been reported to be responsible for acute diarrhea in humans[16,17]. The common major rotavirus genotypes, i.e. G1-G4, G9, P[8], P[4], and P[6], have been found to act as the causative agents for gastroenteritis[18,19]. Recently, the emergence of the novel G12 rotavirus has been detected in different parts of the world[20-26]. A high frequency of G12 associated with multiple *VP4* genotypes has been reported in India, Bangladesh, and Nepal[27-30]. Of all the possible combinations, six genotypes (G1P[8], G2P[4], G3P[8], G4P[8], G9P[8], and G12P[8]) have been observed in 80-90% of the isolated rotavirus infections[31-34].

Both surfaces *VP7* and *VP4* genotypes are able to induce neutralization antibodies and are targets for the vaccine development[15]. Two live, attenuated oral vaccines, Rotarix (GlaxoSmithKline Biologicals, Rixensart, Belgium) and RotaTeq (Merck, Blue Bell, PA, USA) provide protection against severe gastroenteritis caused by the main rotavirus serotypes in circulation and contribute to a significant decrease in diarrhea cases in developed and also in developing countries[33].

Based on data provided by 19 studies in Iran, the prevalence of rotavirus infection has been 36.5% (range 15.3%-67.6%). This pattern is similar to rotavirus infection prevalence in the Eastern Mediterranean region[35]. However in Iran, there is little available information on rotavirus genotype, and limited studies from Iran have reported data on rotavirus G-P combinations. Overall, in Iran, G1P[8], G2P[4], and G4P[8] accounted for over 60% of all rotavirus G-P combinations analyzed[35]. Jalilvand *et al*.[36] evaluated the diversity of *VP7* genes of G1 rotaviruses isolated in Iran. Genetic variations in the *VP7* gene of rotavirus G1 genotype isolates from Iran indicated that rotavirus strains clustered with G1 lineages IA, IC, and IIC. Mapping the rotavirus genotypes distribution will help to determine the epidemiology of the virus strains, especially in different geographic locations. Also, the identification of a novel rotavirus strain may be achieved through a reassortment and interspecies transmission of rotavirus strains from animals to humans. The genotyping of rotavirus is critical to know whether the current vaccines cover the most common genotypes of a certain region[37]. In this research, we aimed to determine the genotype distribution of rotaviruses in children with acute gastroenteritis, admitted to Aboozar Children’s Hospital of Ahvaz, Iran.

## MATERIALS AND METHODS

### Patients and sample collection

For the purpose of a six-month surveillance, after diagnosis of gastroenteritis by a pediatrician, a total of 100 stool samples were obtained from children under five years old with acute diarrhea (diarrhea, vomiting, and abdominal pain less than two weeks) who referred to the Aboozar Children’s Hospital of Ahvaz city (Khuzestan, Iran) during October 2015 to March 2016. Demographic data and clinical signs are presented in Table 1. The children with chronic diarrhea were excluded from the study. Stool specimens were collected and transferred to the clinical laboratory for bacterial culture. All fecal samples were negative for the presence of white blood cells, parasite, and amoeba, and bacterial culture for salmonella, shigella, and other pathogens were transported on ice to the Virology Department and stored at -70ºC for molecular investigations.

**Table 1:** Demographic data and clinical features

Characteristics	Rotavirus infection		*p* value

	Detected in 32 patients (%)	Undetected in 68 patients (%)	
Sex			
Male	59.4	53.1	0.52
Female	40.6	46.9	
Age				
>2 years	21.9	25.0	0.85
<2 years	78.1	75.0	
Symptom				
Vomiting	93.8	60.3	0.00*
Fever ≥38 °C	75.0	52.9	0.01*

* Statistically significant difference

### Antigen detection

All faecal samples were screened for the presence of rotavirus antigen using a commercial latex agglutination kit (Omega Diagnostics Ltd., Omega House, Hillfoots Business Village, Alva FK12 5DQ, Scotland, United Kingdom) according to the manufacturer’s guidelines. Rotavirus antigen-positive fecal samples were stored at -70 °C until use.

### Nucleic acid extraction and cDNA preparation

For those rotavirus antigen-positive samples identified by the latex agglutination test, a faecal suspension of 10% (w/v) was prepared using phosphate-buffered saline, vortexed and centrifuged at 3000 ×g for 15 min. A 200-µl supernatant was collected and used for total RNA extraction using Trizol (Cinagen kit, Iran) according to the manufacturer’s protocol. Following the RNA extraction, 6-µl extracted dsRNA was converted to cDNA with a commercial cDNA Synthesis Kit (Thermo Fisher Scientific, USA). Before the reverse transcription reaction, the RNA samples were incubated at 97 °C for 5 min with *VP6* forward primer, *Vp7* G con reverse primer, and *VP4* con3 forward primer (20 pmol each, 1 µM final concentration). The initial reverse transcription reaction was carried out at 42 °C for 60 min, followed by an inactivation step of 70 °C for 5 min.

### RT-PCR for VP6

Following cDNA synthesis, RT-PCR was performed using specific primer for *VP6* to confirm rotavirus group A (Table 2). The PCR reaction mixture was composed of the 1× PCR buffer (75 mM Tris/HCl [pH 8.8], 1 mM MgCl_2_, 200 µM dNTP mix (Cinagen, Iran), 1 U Taq DNA polymerase (Cinagen, Iran), 1 µl of each primer (10 pmol; Bioneer Company, South Korea), and 2.5 µl of the template. The PCR was performed on peqSTAR 2× (PEQLAB Biotechnologie GmbH, Erlangen, Germany) for 35 cycles. Cycling conditions were as follows: 94 °C for 5 min; 35 cycles at 94 °C for 30 s, 50 °C for 30 s, 72 °C for 1 min, and a final elongation at 72 °C for 10 min. The expected PCR product was 382 bp. The PCR product was subjected to electrophoresis on a 2% agarose gel, stained with a DNA safe stain and observed under ultraviolet light.

**Table 2:** Primers corresponded to VP6, VP7, and VP4 genes for rotavirus genotyping

Primer	Type	Sequence (5′–3′)	Position	PCR product (bp)
VP6-Forward		GACGGV(c)GCR(b)ACTACATGGT	747-766	382[39]
VP6-Reverse		GTCCAATTCATN(d)CCTGGTG	1126-1106	
G-typing (VP7)				
Gcon Reverse		GGTCACATCATACAATTCT	1062-1044	
aBT1	G1	CAAGTACTCAAATCAATGATGG	314-335	749
aCT2	G2	CAATGATATTAACACATTTTCTGTG	411-435	652
Aust	G3	ACGAACTCAACACGAGARG	250-269	813
aDT4	G4	CGTTTCTGGTGAGGAGTTG	480-498	584
aAT8	G8	GTCACACCATTTGTAAATTCG	178-198	885
mG9	G9	CTTGATGTGACTAYAAATAC	757-776	305
P-typing (VP4)				
con3 Forward		TGGCTTCGCTCATTTATAGACA	11-32	
2T-1	P[4]	CTATTGTTAGAGGTTAGAGTC	474-494	484
3T-1	P[6]	TGTTGATTAGTTGGATTCAA	259-278	260
1T-1	P[8]	TCTACTTGGATAACGTGC	339-356	346
4T-1	P[9]	TGAGACATGCAATTGGAC	385-402	392
Universal primer				
VP7con1 Forward		ATGTATGGTATTGAATATACCAC	51-71	1014
VP7con2 Reverse		GGT CAC ATC ATA CAA TTC TAA TC	1062-1040	
VP4con1 Forward		TGGCTTCGCTCATTTATAGACA	2-23	877
VP4con1 Reverse		ATY TCH GAC CAY TTA TAH CC	878-859	

[c = (N = A, T, C or G), b = (R = A or G), d = (Y = C or T)][18]

### Genotyping (G and P) of the strains

Genotyping of samples were conducted according to the WHO manual for rotavirus detection and characterization methods, with the minimal modification of genotyping protocols[38]. A semi-multiplex PCR was employed to amplify the *VP7* and *VP4* genes. For the detection of *VP7* genotypes, following forward primers, aBT1 G1, aCT2 G2, G3-Aust, aDT4 G4, aAT8 G8, mG9 as an Asian Type, and End9 reverse primers for *VP7* were used to be genotyped by semi-multiplex PCR. For the identification of *VP4* genotypes, the appropriate forward and reverse primers were used Table 2).

Typically, the semi-multiplex PCR was performed in a 25-µl volume containing 1× PCR buffer (75 mM Tris/HCl [pH 8.8], 1 mM MgCl_2_, 200 µM dNTP mix) and the appropriate primer mixture (10 pm each and 1 U Taq polymerase). Also, 2.5-µl cDNA was utilized following the thermal-cycling conditions, as described here. The PCR products were electrophoresed on 2% agarose gel, and the G and P genotypes were determined by the sizes of the amplicons (Table 2). Specimens that did not react with either G- or P-type-specific primers were recorded as non-typeable, and cDNA was again synthesized with universal primers (*VP7* con 1F and *VP4* con 1F), and then semi-multiplex PCR was carried under the same condition.

### Sequence analysis for non-typeable strains

A total of 16 PCR products of *VP7* and *VP4* non-typeable strains were sequenced (Bioneer, South Korea). The *VP7* and *VP4* sequences were compared with the sequences retrieved from the GenBank using the online nucleotide BLAST, National Center for Biotechnology Information (NCBI) (http://www.ncbi.nlm.nih.gov/BLAST/).

### GenBank accession numbers

The nucleotide sequence data of gene segments of G12 strains were deposited in the GenBank database and given accession numbers.

### Phylogenetic analysis

To clarify the relationship between the rotavirus isolates, phylogenetic analysis was performed based on *VP7* region. Rotavirus nucleotide sequences were aligned by MUSCLE (Mega 6.0 software). Genetic distances were calculated by the Kimura’s two-parameter substitutions model, and phylogenetic tree was then constructed by the Maximum Likelihood method. The bootstrap probability at a branching point was calculated with 500 pseudo-replicate datasets.

### Ethical consideration

This project (Registration NO.OG-94134) was approved by the ethics committee of Ahvaz Jundishapur University of Medical Sciences (Ahvaz, Iran). All experiments were performed in compliance with relevant laws and institutional guidelines and in accordance with the ethical standards of the Declaration of Helsinki. Stool samples were collected only from those patients who were interested to donate their stool voluntarily. Information pertaining to each patient under the study was kept confidential.

### Statistical analysis

Statistical analysis was performed using the software SPSS, version 19.0. Differences between the groups according to the variables were analyzed using the chi-square (χ^2^) test. *p* values ≤0.05 were considered statistically significant.

## RESULTS

**Fig. 1:**
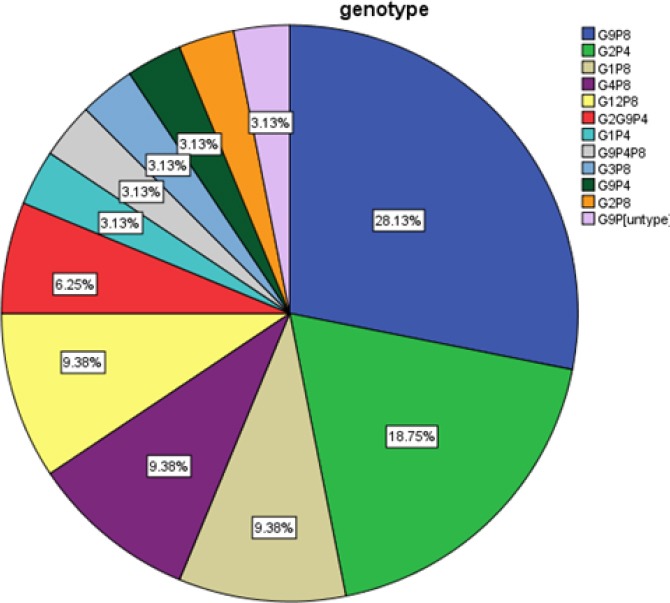
Distribution of human species RVA G/P combinations in children with acute diarrhea

**Fig. 2:**
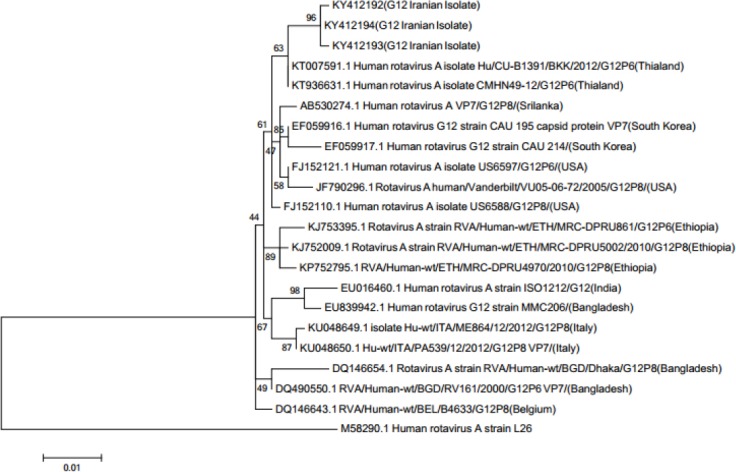
Phylogenetic tree for the G12 VP7 genes from human rotavirus strains available in the DNA databases. The phylogenetic tree was constructed by the Maximum Likelihood method, the genetic distances were computed according to the Kimura 2-parameter model, bootstrap values were obtained after 500 replicate trials, and the VP7 gene of the strain L26 was used as the outgroup.

One hundred faecal samples were collected from 55 (55%) males and 45 (45%) females with acute gastroenteritis during October 2015 to March 2016. The patients’ ages were between 2 and 60 months, with a mean age of 13.40 ± 11.01 months. The rotavirus was detected in 38% (38/100) of patient samples. Six out of 38 (15.78%) rotavirus antigen-positive samples turned out to be negative in the diagnoses of rotavirus by the RT-PCR method. Most rotavirus (10 of 32) detections were observed in children between 6 and 8 months of age. Our analysis of the G and P genotypes of strains from all 32 rotavirus-infected children revealed: 5/32 (15.6%) G type strains and 11/32 (34.3%) P type strains were found non-typeable. Based on the nucleotide sequencing of 1014 bp *VP7* gene fragments and the high degree of identity of the sequences with the GenBank database, 2/5 non-typeable genotypes were identified as G1 and G2 genotypes, and 3/5 non-typeable strains were identified as G12 genotype. All the non-typeable strains for *VP4* were found to be P[8] by nucleotide sequencing of 877-bp *VP4* gene fragments. The rest of the strains were identified as G1-G4, G9, P[4], and P[8] genotypes. The predominant *VP7* genotyping (G type) was G9 (37.5%), followed by G2 (21.9%), G1 (12.5%), G12 (9.4%), G4 (9.4%), G2G9 (6.3%), and G3 (3.1%). As for *VP4* genotyping (P type), P[8], P[4], and P[4] P[8] accounted for 62.5%, 31.3%, and 3.1%, respectively. The distribution of human RVA G/P combinations is shown in Figure 1. Combinations of G typing and P typing were classified as common (G2P[4], G1P[8], and G4P[8]) and uncommon. Common RVA typing was detected in 37.6%. The common and uncommon typing data are shown in Table 3. The predominantly common strains were G2P[4] (18.8%), followed by G1P[8] (9.4%) and G4P[8] (9.4%). Interestingly, the present research indicated a high prevalence (62.4%) of unusual G/P combinations. The predominantly uncommon genotype was G9P[8] (28.1%). Notably, a novel rotavirus strain with G12P[8] that had not been earlier reported in the south of Iran was detected for the first time. Furthermore, the low percentages of mixed infections of 6.3% G types and 3.1% P types were also observed. Only 1 (3.1%) non-typeable (P type) rotavirus strain was detected in this study. Figure 2 shows the nucleic acid identities of the Iranian and other isolates. The GenBank accession numbers for our nucleotide sequences of G12 strains were: KY412192, KY412193, and KY412194.

**Table 3:** Common and uncommon genotyping in infected patients

Genotyping	N	%
Common		
G2P[4]	6	18.8
G1P[8]	3	9.4
G4P[8]	3	9.4
Subtotal	12	37.6
Uncommon		
G9P[8]	9	28.1
G12P[8]	3	9.4
G2G9P[4]	2	6.3
G2P[8]	1	3.1
G3P[8]	1	3.1
G9P[4]	1	3.1
G1P[4]	1	3.1
G9P[4]P[8]	1	3.1
G9P[untype]	1	3.1
Subtotal	20	62.4

N, number

## DISCUSSION

In the present study, almost all known rotaviruses of the G and P types were detected in children with acute gastroenteritis in the Southwest of Iran. Additionally, the emergence of a new G12 type was reported in this study. Rotaviruses are the single most important causes of severe diarrhea in children below five years of age worldwide, accounting for 30% to 50% of the acute diarrheal disease[40]. In the current study, rotavirus infection was detected in one-third of the patients with acute diarrhea (32%). We characterized the *VP7* and *VP4* gene segments and determined the most prevalent rotavirus genotype combinations. The rotavirus genotypes identified were highly diverse. Genotypic variations were further classified as common and uncommon, as described by Kobayashi *et al*.[41]. We found genotype G9P[8], with the prevalence of 28.1%, to be the dominant genotype in the patients studied, while in our previous study, the predominant rotavirus was the G1 genotype, followed by G2[42].

Non-typeable RVAs have been reported in nearly all epidemiologic studies around the world. The reason for the occurrence of non-typeable genotypes is not clear, but it could be due to point mutation at the primer-binding site of the rotavirus gene or to the newly emerging-rotavirus genotypes. A considerable amount of non-typeable genotypes has been reported in studies for G (20.2%; range 5%-41%) and P (17%; range 1.4%-28.2%) genotypes[19]. The most prevalent genotypes, G1P[8], G2P[4], G3P[8], G4P[8], G9P[8], and G9P[6], have been reported in the USA[43]. Similarly, G1P[8] has been reported to be the most frequent genotype in most European countries[34]. Overall, G1P[8], G2P[4], G3P[8], and G4P[8] were the four most common predominant genotypes worldwide[19]. The prevalence of each genotype was 52%, 11%, 3%, and 8%, respectively[19,44]. These results differ from our study and other studies in some parts of the world. Soenarto *et al*. [45] found G1P[6] to be the predominant genotype, but Putnam *et al*.[46] identified G2P [4] genotype as the dominant genotype, followed by G1P[6]. Studies in Romania and Denmark have demonstrated the G9P[8] genotype to be the predominant type, matching the findings of our study and studies from Turkey[47-49]. In Turkey, the most frequent GP combinations were G9P[8] (40.5%), followed by G1P[8] (21.6%), G2P[8] (9.3%), G2P[4] (6.5%), G3P[8] (3.5%), and G4P [8] (3.4%). Although the current study was similar to the Turkey’s study in the G9P8 increasing, there was a difference in the emersion of the G12P8 combination. Ahmed *et al*.[50] in Iraq detected rotavirus in 40% of Iraqi children with acute gastroenteritis. The most prevalent genotype was G2 (40%), most often associated with P[6], followed by G1 (16%), which was mainly associated with P[8] and P[UT]. G3, G4, and G9 were detected at a lower prevalence (3%, 11%, 3%, respectively), mainly related to P[6]. Surprisingly, five G8P[6] and seven G12 RVA strains in combination with P[6] and P[8] were also detected for the first time in Iraq[50]. In addition, in India, Mullick *et al*.[51] have reported G1P[8], G9P[8], G2P[4], and G9P[4][51]. In Japan, Numazaki *et al*.[52] have detected G1P[8], G3P[8], and G9P[8] as the most common genotypes. Kim *et al*.[11], in the Republic of Korea, identified the most common genotypes as G1P[8], G2P[4], and G9P[8] in children with acute diarrhea. In Cameroon, Boula *et al*. [53] reported that G9P[8], G1P[8], and G3P[6] genotypes were the most common genotypes detected in children with acute gastroenteritis. Interestingly, in our study, the emergence of the G12 was found in the G12P[8] combination, whereas the combination of G12P[6] has been detected in the most parts of the world[54]. The G12 genotype was first detected in the Philippines in 1987[55] and then in several other places[56]. In Tehran, Farahtaj *et al*.[57] reported G9P[8] (15.5%) and G12P[8](1.4%), but Shoja *et al*.[35] identified G9P[8] (2.4%) and G12P [8] (0.2%). In the present study, the phylogenetic analyses for *VP7* showed that the *VP7* sequences identified in the Iran/2016 isolates (accession NO. KY412192, KY412193, and KY412194) belong to the G12 cluster statistically supported by 96% bootstrap value (Fig. 2). The similarity of identified nucleic acids among Iranian G12 isolates and Thailand G12 strains were clearly high (~99%). Furthermore, to identify the geographical regions that were the source of the Iranian G12 strain, more samples must be collected from other parts of the country, and a phylogenetic tree needs to be drawn separately. Based on the result of BLASTX, there was 99% amino acid identity among the KY412192 and KY412193 Iranian G12 isolates with the ANJ45307.1 Italian strain. Additionally, the tree indicated that the KY412194 Iranian G12 isolate was 100% identical to ALQ11673.1 isolate from Thailand (Fig. 2).

The phylogenetic analyses for *VP7* by Jalilvand *et al*.[36] showed that nucleotide and amino acid sequences of G1 from Iran had the highest average (93.3% and 95.7%, respectively) of similarity compared with reference sequences of the G1 lineages I and II, However, it demonstrates an average nucleotide and amino acid similarity of 83-92.8% and 90-93.9%, respectively versus all other G1 lineages (III-XI).

Our data are restricted to the prevalence of rotavirus genotypes in the Ahvaz city of Iran. Thus, the prevalence of the rotaviruses genotype including G12 genotype needs to be studied in other parts of Iran. Moreover, the overall prevalence of the G12 rotavirus infection requires to be determined since this study was limited only to hospitalized patients with severe diarrhea. It is now important to compare the antigenic cross-reactivity of the new G12 strains with other common global strains containing vaccine strains. Based on sequence analysis of the *VP7* and *VP4* genotypes described in our study and other reports, distribution of rotavirus genotypes are different in industrialized and developing countries. However, the available data are not sufficient to understand the reasons for this difference. At present, due to the lack of any specific treatment, the rotavirus vaccination program is the only preventive measure against rotavirus infection. Due to the high prevalence of the G9P[8] and G12P[8] genotypes reported in our study and in two studies by Shoja *et al*.[35] and Farahtaj *et al*.[57], it seems that the Rotarix and Rotateq vaccines are ineffective against the mentioned rotavirus genotypes[35,56]. The outlook that rotavirus infection in children may soon be prevented by vaccines has placed a new preference on understanding the diversity of rotavirus strains. The study also emphasizes the coverage of G12 primers for all future rotavirus surveillance.

In conclusion, this study revealed the first detection of the G12 strains in the southwest region of Iran. The most common rotavirus genotype found among the patients was the G9P[8]. All the rotavirus infections occurred in children aged 2-60 months, with peak cases among those of 6 and 8 months of age. These data suggest that G-P genotype combinations vary with regard to geographical locations. This study strongly emphasizes the need for retaining surveillance on emerging rotavirus strains in Iran, and comprehensive investigations are required to determine the prevalence of rotavirus genotypes in other regions of Iran to develop region-specific vaccines.

## References

[1] UNICEF/WHO (2009). Diarrhoea:Why children are still dying and what can be done.

[2] Kosek M, Bern C, Guerrant RL (2003). The global burden of diarrhoeal disease, as estimated from studies published between 1992 and 2000. Bulletin of the world health organization.

[3] Wilhelmi I, Roman E, Sanchez-Fauquier A (2003). Viruses causing gastroenteritis. Clinical microbiology and infectection.

[4] Fodha I, Chouikha A, Peenze I, Beer MD, Dewar J, Geyer A, Messaadi F, Trabelsi A, Boujaafar N, Taylor MB, Steele D (2006). Identification of viral agents causing diarrhea among children in the eastern center of tunisia. Journal of medical virology.

[5] Dung TT, Phat VV, Nga TV, My PV, Duy PT, Campbell JI, Thuy CT, Hoang NV, Van Minh P, Le Phuc H, Tuyet PT, Vinh H, Kien DT, Huy Hle A, Vinh NT, Nga TT, Hau NT, Chinh NT, Thuong TC, Tuan HM, Simmons C, Farrar JJ, Baker S (2013). The validation and utility of a quantitative one-step multiplex RT real-time PCR targeting rotavirus a and norovirus. Journal of virological methods.

[6] Vizzi E, Pineros O, Gonzalez GG, Zambrano JL, Ludert JE, Liprandi F (2011). Genotyping of human rotaviruses circulating among children with diarrhea in Valencia, Venezuela. Journal of medical virology.

[7] Ansari SA, Springthorpe VS, Sattar SA (1991). Survival and vehicular spread of human rotaviruses:possible relation to seasonality of outbreaks. Reviews of infectious disease.

[8] Alexander RG, Romano R (2005). Considerations in creating a beautiful smile. The art of the smile.

[9] Tate JE, Burton AH, Boschi-Pinto C, Steele AD, Duque J, Parashar UD, WHO-coordinated Global Rotavirus Surveillance Network (2012). 2008 estimate of worldwide rotavirus-associated mortality in children younger than5 years before the introduction of universal rotavirus vaccination programmes:a systematic review and meta-analysis. The lancet infectious diseases.

[10] Gautam R, Mijatovic-Rustempasic S, Esona MD, Tam KI, Quaye O, Bowen MD (2016). One-step multiplex real-time RT-PCR assay for detecting and genotyping wild-type group A rotavirus strains and vaccine strains (Rotarix and RotaTeq) in stool samples. Peer journal.

[11] Kim MJ, Jeong HS, Kim SG, Lee SM, Kim SH, Kee HY, Jo Eh, Park Hj, Ha DR, Kim ES, Seo KW, Chung JK (2014). Diversity of rotavirus strain circulated in gwangju, republic of Korea. Osong public health and research perspectives.

[12] Matthijnssens J, Otto PH, Ciarlet M, Desselberger U, Van Ranst M, Johne R (2012). VP6-sequence-based cutoﬀvalues as a criterion for rotavirus species demarcation. Archives of virology.

[13] Ramani S, Liya Hu, Venkataram Prasad BV, Estes MK (2016). Diversity in rotavirus–host glycan interactions:A “Sweet” spectrum. Cellular and molecular gastroenterology and hepatology.

[14] Kudesia G, Wreghitt T (2009). Rotavirus in:Clinical and Diagnostic Virology.

[15] Matthijnssens J, Ciarlet M, McDonald SM, Attoui H, Bányai K, Brister JR, Buesa J, Esona MD, Estes MK, Gentsch JR, Iturriza-Gómara M, Johne R, Kirkwood CD, Martella V, Mertens PP, Nakagomi O, Parreño V, Rahman M, Ruggeri FM, Saif LJ, Santos N, Steyer A, Taniguchi K, Patton JT, Desselberger U, Van Ranst M (2011). Uniformity of rotavirus strain nomenclature proposed by the Rotavirus classification working group (RCWG). Archives of virology.

[16] Matthijnssens J, Rahman M, Martella V, Xuelei Y, De Vos S, De Leener K, Ciarlet M, Buonavoglia C, Van Ranst M (2006). Full genomic analysis of human rotavirus strain B4106 and lapine rotavirus strain 30/96 provides evidence for interspecies transmission. Journal of virology.

[17] Trojnar E, Sachsenroder J, Twardziok S, Reetz J, Otto PH, Johne R (2013). Identification of an avian group A rotavirus containing a novel VP4 gene with a close relationship to those of mammalian rotaviruses. Journal of general virology.

[18] Gentsch JR, Laird AR, Bielfelt B, Griffin DD, Banyai K, Ramachandran M, Jain V, Cunliffe NA, Nakagomi O, Kirkwood CD, Fischer TK, Parashar UD, Bresee JS, Jiang B, Glass RI (2005). Serotype diversity and reassortment between human and animal rotavirus strains:Implications for rotavirus vaccine programs. Journal of infection disease.

[19] Santos N, Hoshino Y (2005). Global distribution of rotavirus serotypes/genotypes and its implication for the development and implementation of an effective rotavirus vaccine. Reviews in medical virology.

[20] Banerjee I, Ramani S, Primrose B, Iturriza-Gomara M, Gray JJ, Brown DW, Kang G (2007). Modification of rotavirus multiplex RT-PCR for the detection of G12 strains based on characterization of emerging G12 rotavirus strains from South India. Journal of medical virology.

[21] Banyai K, Bogdan A, Kisfali P, Molnar P, Mihaly I, Melegh B, Martella V, §Gentsch JR, Szücs G (2007). Emergence of serotype G12 rotaviruses, Hungary. Emerging infectious diseases.

[22] Castello AA, Arguelles MH, Rota RP, Olthoff A, Jiang B, Glass RI, Gentsch JR, Glikmann G (2006). Molecular epidemiology of group A rotavirus diarrhea among children in Buenos Aires, Argentina, from 1999 to 2003 and emergence of the infrequent genotype G12. Journal of clinical microbiology.

[23] Pietruchinski E, Benati F, Lauretti F, Kisielius J, Ueda M, Volotão EM, Soares CC, Hoshino Y, Linhares RE, Nozawa C, Santos N (2006). Rotavirus diarrhea in children and adults in a southern city of Brazil in 2003: distribution of G/P types and finding of a rare G12 strain. Journal of medical virology.

[24] Ramani S, Banerjee I, Gladstone BP, Sarkar R, Selvapandian D, Le Fevre AM, Jaffar S, Iturriza-Gomara M, Gray JJ, Estes MK, Brown DW, Kang G (2007). Geographic information systems and genotyping in identification of rotavirus G12 infections in residents of an urban slum with subsequent detection in hospitalized children:emergence of G12 genotype in South India. Journal of clinical microbiology.

[25] Shinozaki K, Okada M, Nagashima S, Kaiho I, Taniguchi T (2004). Characterization of human rotavirus strains with G12 and P [9] detected in Japan. Journal of medical virology.

[26] Wakuda M, Nagashima S, Kobayashi N, Pongsuwanna Y, Taniguchi K (2003). Serologic and genomic characterization of a G12 human rotavirus in Thailand. Journal of clinical microbiology.

[27] Pun SB, Nakagomi T, Sherchand SB, Pandey BD, Cuevas LE, Cunliffe NA, Hart CA, Nakagomi O (2007). Detection of G12 human rotaviruses in Nepal. Emerging infectious diseases.

[28] Rahman M, Matthijnssens J, Yang X, Delbeke T, Arijs I, Taniguchi K, Iturriza-Gómara M, Iftekharuddin N, Azim T, Van Ranst M (2007). Evolutionary history and global spread of the emerging G12 human rotaviruses. Journal of virology.

[29] Samajdar S, Varghese V, Barman P, Ghosh S, Mitra U, Duttab P, Bhattacharyab SKMV, Panda P, Krishnana T, Kobayashid N, Naik TN (2006). Changing pattern of human group A rotaviruses:emergence of G12 as an important pathogen among children in eastern India. Journal of clinical virology.

[30] Uchida R, Pandey BD, Sherchand SB, Ahmed K, Yokoo M, Nakagomi T, Cuevas LE, Cunliffe NA, Hart CA, Nakagomi O (2006). Molecular epidemiology of rotavirus diarrhea among children and adults in Nepal:detection of G12 strains with P[6] or P[8] and a G11P[25] strain. Journal of clinical microbiology.

[31] Maes P, Matthijnssens J, Rahman M, Van Ranst M (2009). RotaC:a web-based tool for the complete genome classification of group A rotaviruses. BMC microbiology.

[32] Banyai K, Laszlo B, Duque J, Steele AD, Nelson EA, Gentsch JR, Parashar UD

[33] Patel MM, Steele D, Gentsch JR, Wecker J, Glass RI, Parashar UD (2011). Real-world impact of rotavirus vaccination. The pediatric infectious disease journal.

[34] Iturriza-Gomara M, Dallman T, Banyai K, Bottiger B, Buesa J, Diedrich S, Fiore L, Johansen K, Koopmans M, Korsun N, Koukou D, Kroneman A, László B, Lappalainen M, Maunula L, Marques AM, Matthijnssens J, Midgley S, Mladenova Z, Nawaz S, Poljsak-Prijatelj M, Pothier P, Ruggeri FM, Sanchez-Fauquier A, Steyer A, Sidaraviciute-Ivaskeviciene I, Syriopoulou V, Tran AN, Usonis V, Van Ranst M, De Rougemont A, Gray J (2011). Rotavirus genotypes co-circulating in Europe between 2006 and 2009 as determined by EuroRotaNet, a pan-European collaborative strain surveillance network. Epidemiology and infection.

[35] Shoja Z, Jalilvand S, Mokhtari-Azad T, Nategh R (2013). Epidemiology of cocirculating human rotaviruses in Iran. The pediatric infectious disease journal.

[36] Jalilvand S, Afchangi A, Mohajel N, Roohvand F, Shoja Z (2016). Diversity of VP7 genes of G1 rotaviruses isolated in Iran 2009-2013. Infection, genetics and evolution.

[37] Fischer TK, Gentsch JR (2004). Rotavirus typing methods and algorithms. Reviews in medical virology.

[38] World health organization Immunization, vaccines and biological:Strategic Plan 2006-2009.

[39] Iturriza-Gomara M, Wong C, Blom S, Desselberger U, Gray J (2002). Molecular characterization of VP6 genes of human Rotavirus Isolates:correlation of genogroups with subgroups and evidence of independent segregation. Journal of virology.

[40] Bern C, Martines J, de Zoysa I, Glass RI (1992). The magnitude of the global problem of diarrhoeal disease:A ten year update. Bulletin of the world health organization.

[41] Kobayashi N, Ishino M, Wang YH, Chawla-Sarkar M, Krishnan T, Naik TN (2007). Diversity of G-type and P-type of human and animal rotaviruses and its genetic background. Communicating current research and educational topics and trends in applied microbiology.

[42] Azaran A, Makvandi M, Samarbafzadeh A, Neisi N, Hoseinzadeh M, Rasti M, Teymurirad M, Teimoori A, Varnaseri M, Makvandi K (2016). Study on Rotavirus infection and its genotyping in children below 5 years in south west iran. Iranian journal of pediatrics.

[43] Cortese MM, Parashar UD, Centers for Disease Control and Prevention (CDC) (2009). Prevention of rotavirus gastroenteritis among infants and children:recommendation of the advisory committee on immunization practices (ACIP). MMWR recommendations and reports.

[44] Endara P, Trueba G, Solberg OD, Bates SJ, Ponce K, Cevallos W, Jelle Matthijnssens, Joseph N.S (2007). Eisenberg. Symptomatic and subclinical infection with rotavirus P[8]G9, rural Ecuador. Emerging infectious diseases.

[45] Soenarto Y, Aman AT, Bakri A, Waluya H, Firmansyah A, Kadim M, Martiza I, Prasetyo D, Mulyani NS, Widowati T, Soetjiningsih Karyana IP, Sukardi W, Bresee J, Widdowson MA (2009). Burden of severe rotavirus diarrhea in Indonesia. Journal of infection disease.

[46] Putnam SD, Sedyaningsih ER, Listiyaningsih E, Pulungsih SP, Komalarini Soenarto Y, Salim OCh, Subekti D, Riddle MS, Burgess TH, Blair PJ (2007). Group A rotavirus-associated diarrhea in children seeking treatment in Indonesia. Journal of clinical virology.

[47] Anca IA, Furtunescu FL, Pleţca D, Streinu-Cercel A, Rugină S, Holl K (2014). Hospital-based surveillance to estimate the burden of rotavirus gastroenteritis in children below five years of age in Romania. Germs.

[48] Midgley S, Böttiger B, Jensen TG, Friis-Møller A, Person LK, Nielsen L, Barzinci S1, Fischer TK (2014). Human group A rotavirus infections in children in Denmark;detection of reassortant G9 strains and zoonotic P(14) strains. Infection, genetics and evolution.

[49] Durmaz R, Kalaycioglu AT, Acar S, Bakkaloglu Z, Karagoz A, Korukluoglu G, Ertek M2, Torunoglu MA2, Turkish Rotavirus Surveillance Network (2014). Prevalence of rotavirus genotypes in children younger than 5 years of age before the introduction of a universal rotavirus vaccination program:report of rotavirus surveillance in turkey. PLoS one.

[50] Ahmed S, Klena J, Albana A, Alhamdani F, Oskoff J, Soliman M, Heylen E, Teleb N, Tupur Husain, Matthijnssens J (2013). Characterization of human rotaviruses circulating in Iraq in 2008: Atypical G8 and high prevalence of P[6] strains. Infection, genetics and evolution.

[51] Mullick S, Mukherjee A, Ghosh S, Pazhani GP, Sur D, Manna B, Nataro JP, Levine MM, Ramamurthy T1, Chawla-Sarkar M (2014). Community based case-control study of rotavirus gastroenteritis among young children during 2008-2010 reveals vast genetic diversity and increased prevalence of G9 strains in Kolkata. PLoS one.

[52] Numazaki K, Ichikawa M (2014). Clinical and virological characteristics of rotavirus gastroenteritis and prevalence of strains in Tochigi, Japan. In vivo.

[53] Boula A, Waku-Kouomou D, Njiki Kinkela M, Esona MD, Kemajou G, Mekontso D, Seheri M, Ndze VN, Emah I, Ela S, Dahl BA, Kobela M, Cavallaro KF, Etoundi Mballa GA, Genstch JR, Bowen MDK, Ndombo P (2014). Molecular surveillance of rotavirus strains circulating in Yaoundé, Cameroon, September 2007-December 2012. Infection, genetics and evolution.

[54] Ray P, Sharma S, Agarwal RK, Longmei K, Gentsch JR (2007). First detection of G12 Rotaviruses in newborns with neonatal Rotavirus infection at all India institute of medical sciences, new Delhi, India. Journal of clinical microbiology.

[55] Rustempasic SM, Teel EN, Kerin TK, Jennifer Hull J, Roy S, Weinberg GA, Payne DC, Parashar UD, Gentsch JR, Bowen MD (2014). Genetic analysis of G12P[8] rotaviruses detected in the largest U.S. G12 genotype outbreak on record. Infection, genetics and evolution.

[56] Sharma S, Ray P, Gentsch JR, Glass RI, Kalra V, Bhan MK (2008). Emergence of G12 Rotavirus Strains in Delhi, India, in 2000 to 2007. Journal of clinical microbiology.

[57] Farahtaj F, Galimore CI, Iturriza-Gomara M, Taremi M, Zali MR, Edalatkhah H, Fayaz A, Gray JJ (2007). Rotavirus VP7, VP4 and VP6 genotypes co-circulating in Tehran, Iran, between 2003 and 2004. Epidemiology and infection journal.

